# The effect of social distancing on the reach of an epidemic in social networks

**DOI:** 10.1007/s11403-021-00322-9

**Published:** 2021-03-03

**Authors:** Gregory Gutin, Tomohiro Hirano, Sung-Ha Hwang, Philip R. Neary, Alexis Akira Toda

**Affiliations:** 1 Computer Science Department, Royal Holloway, University of London, London, UK; 2 Economics Department, Royal Holloway, University of London, London, UK; 3 College of Business, Korea Advanced Institute of Science and Technology (KAIST), Daejeon, South Korea; 4 Department of Economics, University of California San Diego, San Diego, USA

**Keywords:** SIRwSD model, Social distancing, Infectious subnetwork, WS small-world networks, BA scale-free networks

## Abstract

How does social distancing affect the reach of an epidemic in social networks? We present Monte Carlo simulation results of a *susceptible–infected–removed with social distancing* model. The key feature of the model is that individuals are limited in the number of acquaintances that they can interact with, thereby constraining disease transmission to an *infectious subnetwork* of the original social network. While increased social distancing typically reduces the spread of an infectious disease, the magnitude varies greatly depending on the topology of the network, indicating the need for policies that are network dependent. Our results also reveal the importance of coordinating policies at the ‘global’ level. In particular, the public health benefits from social distancing to a group (e.g. a country) may be completely undone if that group maintains connections with outside groups that are not following suit.

## Introduction

How does social distancing affect the reach of an epidemic such as COVID-19 (Coronavirus Disease 2019) in social networks? To address this question, we consider the idealised problem of the susceptible–infected–removed (SIR) epidemic model (Kermack and McKendrick 1927) in the presence of temporary social distancing constraints placed on the members of a society.

Our *susceptible–infected–removed with social distancing* model (SIRwSD model) is easily understood. As is standard, the vehicle that an infectious disease uses to spread is a *contact network*: a graph, *G*, where vertices represent people and an edge between two vertices *i* and *j* captures the idea that person *i* and person *j* came into contact in such a way that the disease might pass between them.^1^ A social distancing policy is described by a function $\kappa$, defined on the set of vertices of *G*, that constrains the number of neighbours that each person may come into contact with.^2^ At any moment in time, the contact network *G* and social distancing policy $\kappa$ together generate an *infectious subnetwork*. Since the social distancing policy $\kappa$ temporarily deletes a subset of edges from *G*, there are simply less avenues along which the disease may be transmitted.

We run Monte Carlo simulations comparing the reach of the disease when there is no social distancing with that when social distancing measures are imposed.^3^ These comparisons are performed on three types of well-studied networks: the ‘random graphs’ model of Erdős and Rényi (1959) and Gilbert (1959) (hereafter, ERG), the ‘small world’ networks of Watts and Strogatz (1998) (hereafter, WS), and the ‘scale-free’ model of Barabási and Albert (1999) (hereafter, BA).^4^

We begin by focusing on two main questions for the constrained and unconstrained case: (i) What is the likelihood that an infectious disease will become endemic? (ii) What is the distribution of the peak infection rate over the lifecycle of the epidemic? The reason for focusing on (i) is that the level of herd immunity attained is an important policy tool in knowing how, when, and by how much social distancing measures can be relaxed. The reason for focusing on (ii) is that the peak infection rate corresponds to the most overloaded instance that a healthcare service encounters over the lifecycle of an epidemic. Success in both the above dimensions is not simultaneously possible because as one goes up the other goes down. Our results help to guide what the relative trade-offs are.

**Table 1:** Effectiveness of non-pharmaceutical interventions in networks

Network	Variable	$N_{\max }$						
		$\infty$	1	2	3	4	5	10
ERG	${\overline{\mathrm {Peak}}}$	26.5	3.4	7.7	13.9	18.7	19.5	20.7
	Std.($\mathrm {Peak}$)	2.8	7.4	10.7	11.4	8.9	6.1	2.5
	Med.($\mathrm {Peak}$)	26.6	1.0	1.2	20.9	22.0	20.8	20.7
	Removed	87.1	10.2	25.8	50.1	70.1	78.8	83.5
	${\overline{\mathrm {Peak}}}\mid \text {Immunity}$	26.6	25.1	24.7	23.6	22.8	21.1	20.7
	Herd immunity	99.7	10.3	28.2	56.9	80.9	92.4	99.7
WS	${\overline{\mathrm {Peak}}}$	14.5	1.9	3.5	5.1	7.3	8.8	13.3
	Std.($\mathrm {Peak}$)	2.8	3.2	4.8	5.4	5.3	4.6	2.6
	Med.($\mathrm {Peak}$)	14.5	1.0	1.1	1.5	8.9	10.1	13.3
	Removed	82.9	7.5	18.0	29.7	46.3	59.8	81.5
	${\overline{\mathrm {Peak}}}\mid \text {Immunity}$	14.6	12.6	12.6	12.2	11.7	11.3	13.4
	Herd immunity	99.4	7.2	19.9	33.5	54.8	72.1	98.7
BA	${\overline{\mathrm {Peak}}}$	28.7	4.0	8.1	13.8	18.2	18.9	13.0
	Std.($\mathrm {Peak}$)	2.5	8.4	11.4	12.0	9.3	6.2	2.6
	Med.($\mathrm {Peak}$)	28.7	1.0	1.2	20.3	21.6	19.9	12.7
	Removed	80.7	10.4	23.6	43.3	60.9	69.8	66.1
	${\overline{\mathrm {Peak}}}\mid \text {Immunity}$	28.7	27.1	26.2	24.6	22.8	20.4	14.1
	Herd immunity	99.9	11.5	28.2	54.1	78.6	89.7	49.2

${\overline{\mathrm {Peak}}}$” is the mean peak infection rate (%). ‘Std.’ and ‘Med.’ are the standard deviation and median of peak infection rates across 1000 simulations. ‘Removed’ is the fraction (%) of population removed (recovered) by the end of the epidemic. ‘${\overline{\mathrm {Peak}}}\mid \text {Immunity}$’ is the mean peak infection rate conditional on acquiring herd immunity. ‘Herd immunity’ is the fraction of simulations (%) in which herd immunity was acquired

While the spread of an epidemic is curtailed when all individuals in a society face the same constraints, the reduction varies greatly depending on the topology of the social network. For all three social network structures, ERG, WS, and BA, that we consider, strong measures of social distancing (limiting everyone’s daily interactions to 3 or fewer) stops an epidemic with high probability (see Fig. 3). However, for societies structured according to WS, the fraction of individuals who are in state *R* (‘removed’) after the epidemic has passed is much less over this range (see rows labelled ‘Herd Immunity’ in Table 1). Moderate social distancing (defined as 4–5 interactions per period) delays the peak of an epidemic but has little effect on the size of the peak and on the number that the disease ultimately reaches. The effect of mild social distancing (limiting individuals to 10 social interactions) differs greatly across network structures. With ERG networks, the effect is negligible. With WS networks, the outcome is the same as no intervention. With BA networks, the peak is significantly reduced.

While we begin with comparing the outcomes of no social distancing with those to social distancing, our framework is flexible enough to address a host of other policy experiments. For example, we consider a network comprised of two densely connected components, interpreted as ‘countries’, that have a small number of connections between them that we interpret as international friendships. We show that the public health benefits to a country that imposes strong social distancing measures are dramatically reduced, and perhaps eradicated entirely, if that country continues to allow international connections, interpreted as maintaining open borders, with a country that is not implementing similar measures. In a hyper-connected world, this points to the need for ‘global’ cooperation to eradicate an epidemic. In particular, if the global approach is uncoordinated with each country unilaterally applying social distancing measures without taking into account the policy choices of its neighbours, then an infectious disease may cycle around for far longer than otherwise desired.

We also consider what happens when a subset of individuals are deemed ‘essential workers’ who can go about their lives facing weaker social distancing constraints than the rest of the population. Our results show that even if only a small fraction of the population is deemed essential, the reach of the epidemic is similar to that wherein there are no essential workers. Finally, we consider another policy tool: time. Specifically, we consider a policy that begins with severe social distancing measures that are incrementally relaxed over time, and compare the outcome with that from a policy of mild but constant social distancing over a shorter window. We find that the public health outcome is better under gradual relaxation for ERG networks and WS networks, but worse for BA networks.

We conclude the paper by discussing how it fits in the literature and by suggesting some extensions to our framework that can be implemented in future work. However, we emphasise that the framework of this paper considers the benefits to social distancing when it is the only policy tool available. Clearly, this is not realistic. In tackling real-world epidemics, policy makers have an array of tools available. For practical purposes, governments need to understand how the many different epidemic management policies complement each other. This paper is about one such policy tool in isolation, but given its tractability, we hope that it can be incorporated into richer models.

## The model

Modelling how an infectious disease might spread through a population is done using a *contact network*: an undirected graph, $G = (V, E)$, where *V* is the set of vertices, and *E* is the set of edges. Vertices represent individuals and an edge between two distinct vertices *i* and *j* captures the idea that these two people are acquaintances and meet in such a way that the disease may be transmitted from one to the other.

We assume throughout that *G* is connected. The neighbourhood of vertex *i* in *G* is denoted *N*(*i*) and its degree is denoted $d(i) = |N(i)|$. The *capacity* of *G* is a function $\kappa : V \rightarrow \mathbb {Z}_{\ge 0}$. This capacity function $\kappa$ is our measure of social distance. A contact network *G* and social distancing policy $\kappa$ together generate an *infectious subnetwork*, $G^{\kappa }$. Intuitively, the policy $\kappa$ restricts the number of neighbours that each vertex can interact with, which will in turn cap the number of neighbours to whom any infected person can transmit the disease in a given period.^5^

Our *SIRwSD* model then operates as follows. Everyone in the population is currently in one of three states: *susceptible* (*S*)—has not had the disease and is therefore at risk; *infectious* (*I*)—currently has the disease and may therefore pass the disease to others; *removed* (*R*)—has had the disease and is no longer infectious (may be immune, isolated, dead, etc.). Time is discrete, starts at $$t = 0$$, and goes forever. Let $$S_{it}$$, $$I_{it}$$, $$R_{it} \in \left\{ 0, 1\right\} $$ be the status of individual *i* at time *t*, where 1 means being that status and 0 means not being that status. (Clearly, $$S_{it} + I_{it} + R_{it} = 1$$ for all $$i \in V$$ and all times *t*.) In every period, each infected vertex *i* randomly selects $$\kappa (i)$$ of its neighbours, and, if any of the selected neighbours are in state *S*, then they become infected with probability $$\beta \in (0, 1)$$.^6^ The probability of removal (moving from state *I* to state *R*) is denoted by $$\gamma \in (0, 1)$$. We assume that $$\gamma $$ is constant over time and is the same for everyone. Once an individual enters state *R*, he stays there forever more. We say that the system has *stopped* when there is no individual in state *I*.^7^$$^{,}$$^8^

**Fig. 1:**
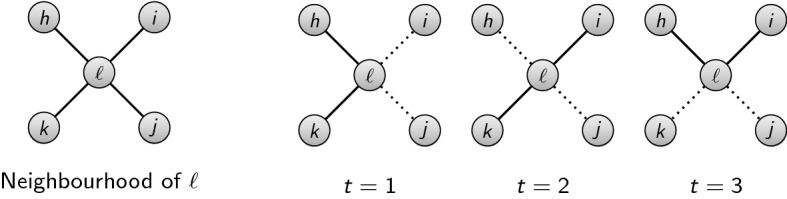
The social distancing policy $N_{\max }= 2$ in effect for three periods

To illustrate how social distancing works in practice, we refer to Fig. 1. This figure can be thought of ‘zooming in’ on the local neighbourhood of individual $\ell$ who has 4 neighbours in the contact network. The leftmost image shows precisely this. The next three images illustrate what transpires when a social distancing policy with $\kappa (\ell ) = 2$ is imposed for three periods, with period $t=1$ being the first period in which the policy takes effect. In each period $t = 1, 2, 3$, individual $\ell$ randomly selects two of their four neighbours to interact with. The interactions that occur are depicted by solid lines while those that don’t occur are depicted by dashed lines. For the realisation depicted, $\ell$ interacts with neighbours *h* and *k* in period 1, with neighbours *i* and *k* in period 2, and with neighbours *h* and *i* in period 3. Note that $\ell$ never encounters neighbour *j* under this realisation. Thus the disease being transmitted (directly) from $\ell$ to *j* is impossible while social distancing is in effect but would be possible without.

It is now straightforward to visualise how the SIRwSD model operates by extrapolating from what transpired in the neighbourhood of $\ell$ in Fig. 1 to the entire contact network *G*. Assume the social distancing policy is given by $\kappa$, that it starts in period 1 and is in effect for *T* periods. Denote the infectious subnetwork in period $t \in \left\{ 1, \ldots , T\right\}$ by $G^{\kappa }_{t}$. For the duration of the policy, the capacity constrained SIR model is simply an SIR model operating on the sequence of infectious subnetworks $\left\{ G^{\kappa }_{1}, G^{\kappa }_{2}, \ldots , G^{\kappa }_{T}\right\}$.^9^

This concludes the discussion of our SIR with Social Distancing model. Since the state of the system at any time *t* is given by $\left\{ (S_{it}, I_{it}, R_{it})\right\} _{i \in V}$, all that is required to run simulations are to specify the environment, social distancing policy, and initial condition. This requires disease parameters $\beta$ and $\gamma$, a graph *G*, a sequence of capacity functions $\left\{ \kappa _{1}, \kappa _{2}, \ldots , \kappa _{T}\right\}$, and initial condition $\left\{ (S_{i0}, I_{i0}, R_{i0})\right\} _{i \in V}$. With all this, the capacity constrained SIR model is a well-defined stochastic process that is easily simulated.

## Simulation parameters

We run simulations on the capacity constrained SIR model described above for three different types of graphs: ERG, WS, and BA. We model one period as a day to calibrate network and epidemic parameters.

### Network parameters

We briefly review how each kind of graph is generated and the graph-parameters required to render them like-for-like. (See Footnote 4.)

Erdős and Rényi (1959) and Gilbert (1959) These networks are often referred to simply as ‘random graphs’. Begin with the *n* vertices and an empty edge set. Now, for every pair of distinct vertices *i* and *j*, form edge *ij* with fixed probability *p*. The expected number of edges in the resulting graph is $\frac{1}{2}n(n-1)p$, because there are $$\left( {\begin{array}{c}n\\ 2\end{array}}\right) =\frac{1}{2}n(n-1)$$ potential edges and each edge is realised with probability *p*.

Watts and Strogatz (1998) The resulting network is often referred to as a ‘small world’. Begin with the *n* vertices connected in a ring lattice where each vertex has *k* neighbours to the left and *k* neighbours to the right, where $k \ll n$. Proceed clockwise around the ring one time, and for each vertex, rewire every edge that it has with the *k* vertices immediately to its right with rewiring probability *q*. Note that since the net change in the number of edges is zero, the number of edges in the resulting graph remains exactly *nk*, because each vertex has 2*k* neighbours and each edge is counted twice.

Barabási and Albert (1999) The resulting network is often referred to as ‘scale free’. Begin with a complete graph on $m_0 \ll n$ vertices and allow time to increment forward from $t=1$ to $t = n-m_{0}$. At each point in time, a new vertex is born and the newly born vertex forms one edge with $m \le m_{0}$ of the existing vertices, where the probability that the newly born vertex connects to existing vertex *i* is given by $d(i)/\sum _{j}d(j)$. In total $n-m_{0}$, new vertices are added so the number of edges in the resulting graph is always exactly $\frac{1}{2}m_0(m_0-1)+m(n-m_0)$.

We choose to equalise average degree, ${\bar{d}}$, across all network types. This ensures we are comparing like-for-like since it is the way in which the societies are organised, and not the overall level of interaction, that is being varied. Given that the number of edges in each graph will be $n{\bar{d}}/2$, some straightforward algebra yields the following parameter requirements: $p = \frac{{\bar{d}}}{n-1}$ for ERG, $k=\frac{{\bar{d}}}{2}$ for WS, and $m=\frac{n\bar{d}-m_0(m_0-1)}{2(n-m_0)}$ for BA.

In each network, we fix the number of vertices to be $n =1000$ and set $\bar{d} = 10$.^10^ Such a choice requires setting the ERG parameter $p = 0.01$. For WS, we choose rewiring probability $q = 0.1$, which is relatively standard, and $k = \bar{d}/2 = 5$. For the BA network, for simplicity we set $m_{0} = 1$, and round up *m* so that $m=\bar{d}/2=5$.

### Epidemic parameters

In the absence of a cure, the removal probability $\gamma$ is a biological parameter determined by the infectious disease. Through contact tracing in Wuhan, China, Li et al. (2020) estimate the mean serial interval for COVID-19, which corresponds to $1/\gamma$, to be 7.5 days. We round $\gamma$ to the first significant digit and set $\gamma =0.1$. To calibrate $\beta$, we proceed as follows. Since average degree is $\bar{d}$, an infected individual will infect $\beta \bar{d}$ others in one period and on average $\beta \bar{d}/\gamma$ over the infectious period when social distancing is unconstrained. The expression $\beta \bar{d}/\gamma$ must equal the basic reproduction number ${\mathcal {R}}_0$ of the infectious disease, which is estimated to be around 3 in Toda (2020) for COVID-19.^11^ Given our choice of $\bar{d}=10$ and $\gamma =0.1$, we have that $\beta = 0.03$. Finally, we initialise the system by setting each vertex to state *I* with probability $y_{0} = 0.01$ (so that, on average, each trial starts with 1% of the population infected).

To verify that our calibration of the transmission probability $\beta$ is correct, we simulate the effective reproduction number ${\mathcal {R}}_t$ in our social network model. In the classical SIR model of Kermack and McKendrick (1927) (in continuous-time and homogeneous interaction), the evolution of the fraction of infected agents $y_t$ satisfies the differential equation

1 $$\begin{aligned} {\dot{y}}_t=\beta x_ty_t -\gamma y_t=\gamma (\beta x_t/\gamma -1)y_t=\gamma ({\mathcal {R}}_t-1)y_t, \end{aligned}$$

where $\beta >0$ is the transmission rate, $\gamma >0$ is the recovery rate, $x_t\in [0,1]$ is the fraction of susceptible agents, and ${\mathcal {R}}_t=\beta x_t/\gamma$ is called the effective reproduction number. Dividing both sides of (1) by $y_t>0$, after some algebra we obtain

2 $$\begin{aligned} {\mathcal {R}}_t=1+\frac{1}{\gamma }\frac{\mathrm {d}}{\mathrm {d}t}\log y_t. \end{aligned}$$

Using the analogy from (2), in our discrete-time model, we may *define* the effective reproduction number by

3 $$\begin{aligned} {\mathcal {R}}_t=1+\frac{1}{\gamma }(\log y_t-\log y_{t-1})=1+\frac{1}{\gamma }\log \frac{y_t}{y_{t-1}}. \end{aligned}$$

**Fig. 2:**
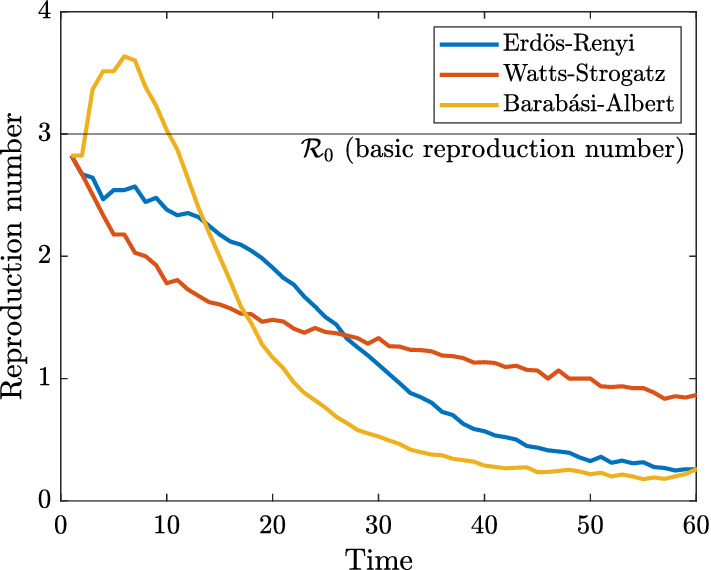
Median effective reproduction number

Figure 2 plots the median effective reproduction number (3) across 1000 simulations described below for each network type. Consistent with our construction, the simulated effective reproduction number is close to the theoretical value ${\mathcal {R}}_0=3$ at the beginning of the simulation. Therefore, we confirm that we are comparing networks like-for-like.^12^

## Simulation results

While a social distancing measure $\kappa$ may assign individual-specific constraints, in this section we will assume that it does not.^13^ This allows us to abuse notation somewhat by simply writing $N_{\max }$ for the maximum number of neighbours that each individual will interact with.^14^

**Fig. 3:**
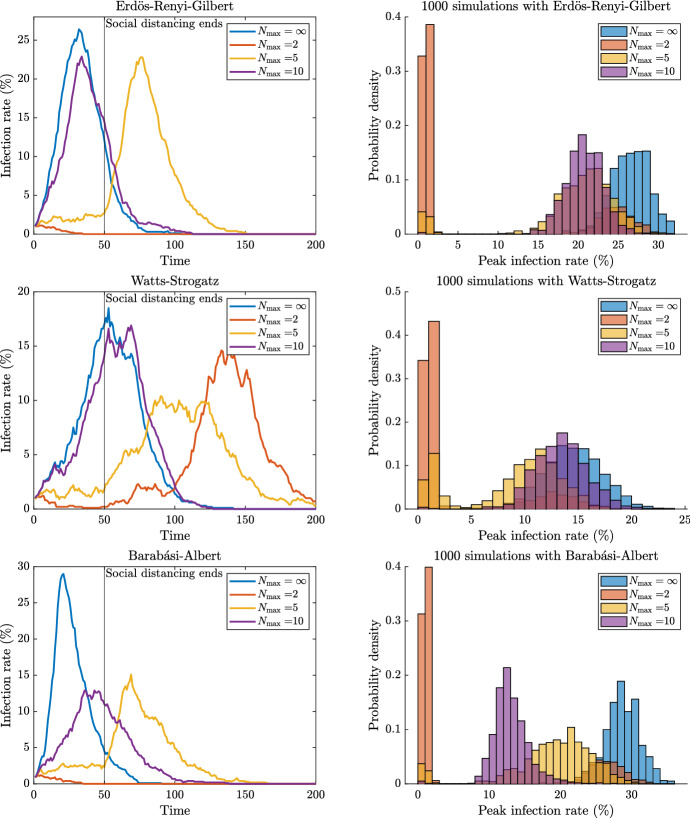
Results for one trial (left) and 1000 trials (right)

We allow the social distancing measure $N_{\max }$ to take values in the set $\left\{ 1, 2, 3, 4, 5, 10, \infty \right\}$, where the value $\infty$ denotes the standard SIR model (i.e. our model without any constraints). Lastly, unless otherwise stated, we suppose that the social distancing measure starts in period 1 and is lifted from period 51 onwards. Figure 3 contains six panels organised in a $3\times 2$ format. The first row refers to ERG networks, the second to WS networks, and the last row to BA networks.

The left panel in each row presents results of four single trials where the trials are distinguished by the value of $N_{\max }= 2, 5, 10$, and $\infty$ (we choose not to present all values of $N_{\max }$ as the resulting image is too cluttered). The horizontal axis is time and the vertical axis is infection rate in the population. The vertical line at $t=50$ represents the lifting of the social distancing restriction.

The right panel in each row presents a histogram of peak infection rates computed from 1000 simulations of the type shown in the left panel. To see the connection between the two panels, we note that each left panel provides one data point for the right panel. To further cement understanding, note that in the left panel of the WS row, the peak infection rate for $N_{\max }= 2$ trial is greater than that for the $N_{\max }= 5$ trial.

We begin with the left panels referring to individual trials. For the ERG and BA networks, the social distancing measure of $N_{\max }= 2$ eradicates the epidemic within the 50-period window. Note however that the $N_{\max }= 2$ trial does not eradicate the epidemic for the WS network. For each of the three network structures, the trial with moderate social distancing, $N_{\max }=5$, delays the peak of the epidemic but has little impact on the size of the peak. The effect of mild social distancing, $N_{\max }=10$, differs across network structures. With a random network (ERG), the effect is mild. With a small-world network (WS), the outcome appears equivalent to no intervention ($N_{\max }= \infty$). This is because in the WS network, each agent has $\bar{d}=10$ neighbours, so the restriction $N_{\max }=10$ does not bind. (The histograms differ only due to sampling error.) With a power law network (BA), the peak is significantly reduced. This is because in the BA network there are a small number of individuals with very high degree. So, since many of the edges of the contact network have a high degree individual at one end, the same holds true for the infectious subnetwork regardless of what it may be. This means that these ‘hubs’ are very likely to become infected even under social distancing. However, due to social distancing, an infected hub infects nowhere near as many as when there are no constraints. These hubs no longer act as ‘super-spreaders’.

While the findings in the left panel of Fig. 3 are illustrative, they represent only a single trial and may not be representative. As such, to evaluate the robustness, we now turn our attention to the panels in the right-hand column. Here, each panel presents histograms of the peak infection rate attained in 1000 trials for a given network type (four histograms on each graph—one for each of the same measures of social distancing as in the left panels).^15^ For all three network structures, setting $N_{\max }=2$ eradicates the disease in $\sim 70\%$ of the trials. We note that the empirical frequency distribution of peak infection is bimodal for the value $N_{\max }= 2$ with the lower mode corresponding to the epidemic being eradicated. Setting $N_{\max }=5$ eradicates the disease in slightly more than $\sim$ $10\%$ of the trials of the WS networks and even less for ERG and BA. Setting $N_{\max }= 10$ almost never eradicates the disease for any kind of network. Setting $N_{\max }= \infty$ never eradicates the disease for any kind of network.

Conditional on the disease not being eradicated, the distribution of peak infection rate is the same as no intervention for ERG and WS. This is because the epidemic restarts after the lifting of social distancing measures. However, for the BA network, conditional on the disease not being eradicated the distribution of peak infection rate for $N_{\max }= 5$ is sandwiched between that of $N_{\max }= 10$ and $N_{\max }=\infty$. At first glance, this may appear mysterious but in fact it is not. The explanation is that the distribution of peak infection rate presented says nothing about the precise moment, during a particular trial, that the peak infection rate was attained. For $N_{\max }= 5$, the peak infection rate will typically occur *after* the social distancing restrictions have been lifted (as can be seen for the trial in the left panel of ERG), but this is not the case for $N_{\max }= 10$. For BA networks, it would appear that setting $N_{\max }= 10$ is a superior policy to the policy to setting $N_{\max }= 5$.

Table 1 presents further results. The variable ‘$\mathrm {Peak}$’ denotes the peak infection rate. ‘Std.’ and ‘Med.’ denote its standard deviation and median, respectively. ‘Removed’ denotes the fraction of population that was removed (has recovered) by the end of the epidemic, which also equals the cumulative number of infections. Letting *T* denote the last period before the system stops, we say that the society has acquired *herd immunity* if $\beta \bar{d} x_T\le \gamma$, where $x_T$ is the fraction of susceptible individuals at time *T*. This condition roughly says that a new infection no longer causes an exponential growth in cases. (See Toda 2020 for a discussion.) The variable ‘Herd immunity’ denotes the fraction of simulations in which herd immunity thus defined has been acquired.

As can be seen from Table 1, there is a trade-off to be made between acquiring herd immunity and keeping the peak low. In most specifications, the probability of acquiring herd immunity and the average peak are both increasing in $N_{\max }$. This suggests that drastic social distancing policies are fragile in the sense that while they tend to contain the disease, it is less likely to achieve herd immunity and the society is susceptible to a recurrent epidemic. Furthermore, note that the variable ‘${\overline{\mathrm {Peak}}}\mid \text {Immunity}$’ (meaning ‘average peak conditional on herd immunity being attained’) is decreasing in $N_{\max }$. This shows that when the disease control has failed, the peak is actually worse (higher) under more strict social distancing policies.

Perhaps the most surprising result in Table 1 is the value of the variable ‘Herd immunity’ for BA networks when $N_{\max }=10$. The value it takes is 49.2, which is considerably less than the value it takes when $N_{\max }=5$. The reason for this is that when $N_{\max }=10$, the potential ‘super-spreaders’ (those with very high degree) are more likely to become infected during the social distancing window than when $N_{\max }=5$. But given the vertices with high degree become infected during the measures, they are likely to be recovered when the social distancing restriction is lifted. As such, these vertices do not maximise their reach.

In summary, choosing a small $N_{\max }$ such as $N_{\max }=1,2$ reduces the peak but prevents building herd immunity, which makes the society susceptible to further epidemics. Choosing an intermediate $N_{\max }$ such as $N_{\max }=3,4,5$ does not necessarily reduce the peak, while mildly preventing herd immunity. Choosing $N_{\max }=10$ generally reduces the peak infection rate *and* achieves herd immunity.

## Policy issues

Our set-up is sufficiently flexible that we can address a wide range of policy issues. In this section, we discuss some extensions and present the results.

### Tackling epidemics requires ‘global’ cooperation

Suppose there are two countries, Country *A* and Country *B*. Each country has a population of *n* individuals, and when viewed in isolation each country forms its own connected network. Suppose further that every pair of individuals from different countries are randomly connected with fixed probability 1/(10*n*), and we interpret connections of this form as international relationships. (We choose a low value so that in expectation only a very small minority of each individual’s interactions are with foreigners.)

**Fig. 4:**
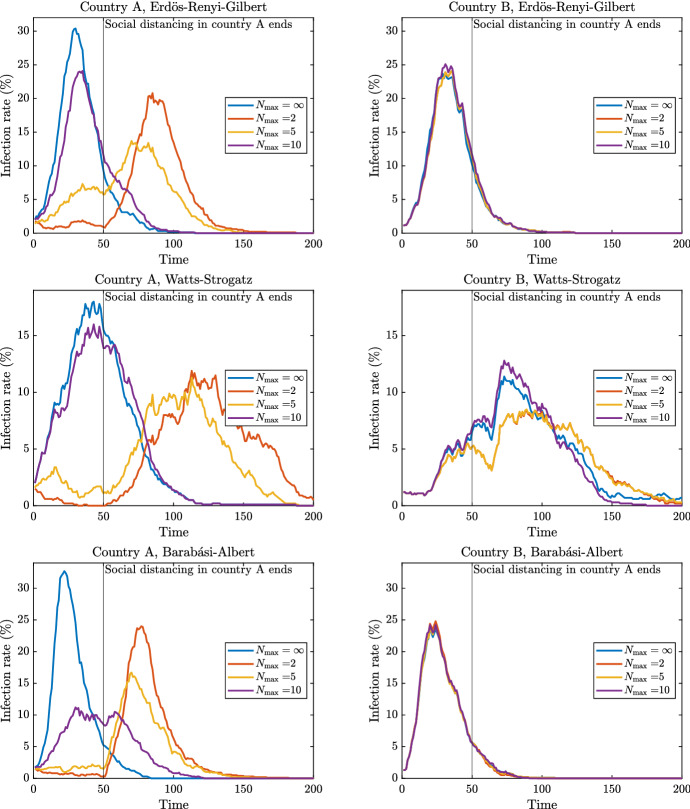
Epidemic dynamics with two countries

For the simulations, we set $n=1000$ and $\bar{d} = 10$ for each country, and we assume that both countries have the same social structure (both ERG, both WS, or both BA). We use the same values for $\beta$, $\gamma$, and initial infection probability $y_{0}$, as before.^16^ However, instead of assuming that temporary social distancing measures are imposed on everyone as in the simulations of Sect. 4, we now suppose that Country *A* levies social distancing measures while Country *B* imposes no measures of any kind. Figure 4 depicts the simulation results for one trial of the infection rate in both countries as a function of time for the range of different social distancing measures in Country *A* given by $N_{\max }= 2, 5, 10$, and $\infty$.

Figure 4 contains six panels organised in a $3\times 2$ format. As with Fig. 3, the panels in a given row correspond to different network structures: the first refers to ERG networks, the second to WS networks, and the last row to BA networks. Within a given row, the left panel presents time-varying infection rates for Country *A* of four separate simulations differing across social distancing measures ($N_{\max }= 2, 5, 10$, and $\infty$). The vertical line at $t=50$ represents the lifting of the social distancing restriction. The right panel in each row is the corresponding time-varying infection rate for Country *B* during the same simulation.

The interesting comparison to make is the results for Country *A* above with that of the single country results in the left panels of Fig. 3. Compared to the case with one country, it is clear that social distancing is less effective because new cases are imported from foreign countries. And this holds even for the strict nearly-full-lockdown social distancing measure of $N_{\max }= 2$. Note that this occurs despite the fact that the expected degree for vertices in Country *A* has increased by the seemingly negligible amount of 0.1.

These simulation results highlight the need for policies designed to tackle the COVID-19 epidemic to be coordinated at the global level. To give an extreme example, imagine a large body of interconnected individuals who live in different regions with no barriers to moving between regions (this is precisely with countries within the Schengen area of the European Union and states within the USA). If one region has weaker social distancing measures than all the others while maintaining connections to them, this one region can impose large negative externalities on the others, perhaps even completely wiping out the benefits one region’s strict measures.

### Essential workers

Suppose that some fraction of individuals are deemed ‘essential workers’ who face different social distancing measures to everyone else. As a numerical example, suppose a random 10% of the individuals in the population are essential workers, and the social distancing policy is far more lax for these individuals, specifying $N_{\max }=10$ over the entire duration.

**Fig. 5:**
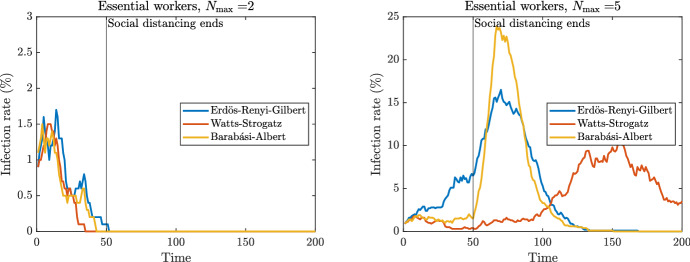
Epidemic dynamics with essential workers

Figure 5 contains two panels. In both panels, 10% of individuals are randomly designated as essential workers and assigned $N_{\max }= 10$. The left panel shows the outcomes of one trial for each network type, wherein 1% of all individuals are initially infected and the 90% of individuals who are non-essential have $N_{\max }= 2$. The right panel is similar except the 90% of individuals who are non-essential have $N_{\max }= 5$.

In comparing the graph from each panel with the corresponding appropriate panel from Fig. 3, it is clear that there is only a minor difference from the benchmark case where there are no essential workers. As such, if only social distancing measures were employed, they would not be very effective in curtailing the spread of the disease unless extremely strict measures are imposed on those who are deemed not essential. This seems highly relevant to the COVID-19 pandemic given that in a recent paper, McCormack et al. (2020) used the Public Use Microdata Sample of the 2018 American Community Survey (ACS) to estimate that one in every three jobs in the USA is deemed essential.

### Gradual relaxation of social distancing

In tackling COVID-19, many countries introduced a ‘tier structure’ of measures, where higher tiers correspond to stricter social distancing. This raises two natural policy issues: (i) How many tiers should there be? (ii) When should a region be permitted/forced to move down/up a tier? While the second policy allows governments to respond in real time in the event of getting a bad draw, the first policy of where to place the various tiers needs to be decided in advance. Implicitly, both these policies are exploiting time-varying mitigation strategies.

As an example, we consider the outcomes of two different ‘tiered’ policies. They are,

Policy A: Start with severe social distancing, $N_{\max }= 2$, for 10 days, then increment $N_{\max }$ by 1 every 10 days stopping when $N_{\max }= 10$. After this 90-day window, all social distancing restrictions are lifted.
Policy B: Start with mild social distancing, $N_{\max }= 10$, and keep in place for 50 days. After this 50-day window, all social distancing restrictions are lifted.

**Fig. 6:**
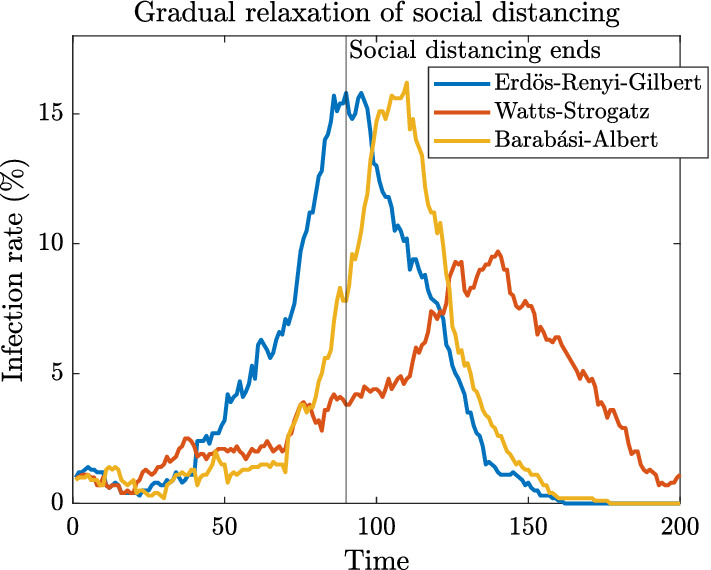
Epidemic dynamics with gradual relaxation of social distancing

Policy B above is identical to that which has already been considered (the results can be seen in Fig. 3 and Table 1). Figure 6 presents simulation results from one trial of Policy A for each network type.

At least for this trial, setting $N_{\max }=10$ for 50 days is considerably better for BA networks, while gradual relaxation over 90 days is better for ERG and WS. The reasons for this are similar to the results of Sect. 4. That is, for BA networks Policy A has the important effect of infecting the well-connected individuals but constraining their reach during their infectious period and this trumps the outcome where they are not infected until after restrictions are lifted.

## Extensions and related work

Our goal with this paper is to provide a flexible framework that allows policy makers to simulate precisely how social distancing can mitigate the spread of an infectious pathogen. The model we have proposed is the standard SIR model on a network with social distancing constraints imposed upon individuals, that we call the SIRwSD model. While we have considered some such social distancing policies in Sect. 5, there are of course many more. We briefly sketch some of these now.

**Other social distancing models.** Perhaps the paper closest to ours is Maharaj and Kleczkowski (2012). These authors consider a networked SIR model on a square lattice. They introduce two radii: the first radius defines an individual’s neighbourhood and the second radius defines the individual’s neighbourhood with social distancing measures in effect. The main differences with our model and this is that we allow individual-specific social distancing restrictions, and we do not limit analysis to networks where all individuals are in some sense ‘the same’.

**Random interactions.** Even during a full lockdown, setting $\kappa (i) = 0$ for every individual *i*, people are still permitted to make outings for essential items. As such, there is always the possibility that an individual will contract a disease from someone with whom they are not social acquaintances. This feature could easily be included in the model by allowing the possibility of interactions that are not specified by the contact network.

**Asymmetries in transmission probabilities.** We have assumed that the transmission probability $\beta$ is the same for all individuals. But there is medical literature that suggests that this is not so for COVID-19 (Iype and Gulati 2020; Zhou et al. 2020). We have further assumed that the recovery probability $\gamma$ is constant for everyone. But, at least as regards deaths attributed to the COVID-19 pathogen, this is not accurate, with the likelihood of recovery decreasing with age.^17^ Both of these richer features can easily be incorporated into our set-up.

**Directed connections.** Most models of how epidemics spread in social networks assume the underlying graph is undirected. This implies that the individuals most likely to spread the disease are also the most likely to become infected. Allard et al. (2020) relax this assumption allowing the underlying graph to be directed (and not just a complete biorientation of an undirected graph, so that the in-degree of a vertex need not equal its out-degree). Allard et al. (2020) show that this can have policy implications for both contact tracing and the prevention of super-spreading events. Our social distancing measure can easily be appended to a ‘directed’ framework by constraining each vertex *v* according to the arcs with tail at *v* (the out-edges of vertex *v*).

**Reinfection is possible.** The *R* state in the SIR model refers to ‘Removed’, but is more accurately described as ‘Removed and no longer Susceptible’. As regards COVID-19, there is new data emerging from South Korea that indicates that some who have been infected may be prone to reinfection. If so, the spread of the coronavirus may be better described by an SIS model (where the second ‘S’ also stands for susceptible). This important change is easily incorporated to our set-up.

**Asymmetries in global travel and ‘Travel Corridors’.** Connections between counties need not always be equally distributed as we assumed in Sect. 5.1. For example, the number of UK citizens who holiday in Spain dwarfs the number of Spanish citizens who holiday in the UK. Such a set-up could be incorporated into our framework by assuming that a large number of UK citizens (the holiday makers) each have a few connection to a small number of Spanish citizens (those who work in hospitality). How such asymmetries in connections play out may be important in deciding which ‘travel corridors’ are safer than others.^18^ Such policies are easily explored using our framework.

**Individual-specific policies: key to target the right individuals.** As mentioned before, a contact network *G* and social distancing policy $\kappa$ together generate an infectious subnetwork. Since the social distancing policy $\kappa$ temporarily deletes a subset of edges from *G*, there are simply less avenues along which the disease may be transmitted. However, perhaps counterintuitively this will not always reduce the reach of an epidemic. While our results in Sect. 4 show that the reach is reduced when all individuals in society face the same constraints, Muscillo et al. (2020) show that if those individuals who have many neighbours reduce their contacts proportionately less than those who have few, then the disease can take longer to die out. The specification considered by Muscillo et al. (2020) is a special case of our framework where $\kappa$ is chosen such that the fraction $\kappa (i)/d(i)$ is increasing in degree.

**Interplay between time-varying and individual-specific constraints.** In Sects. 5.1 and 5.2 , we allowed social distancing constraints to be group-specific (i.e. varying from individual to individual). In Sect. 5.3, we considered time-varying policies. How group-specific constraints and time-varying constraints interact together is not something we have explored in this paper but can easily be handled within our framework.

**Full compliance and rational individuals.** In our model, a social distancing policy is set and all individuals adhere to it. Clearly, this a strong assumption as full compliance cannot be guaranteed without strong monitoring. We can easily incorporate this feature in our model is by defining a parameter $\xi \in (0, 1)$ where $\xi$ is the probability that an individual violates the restrictions in a given period. A related point is made by the game-theoretic literature on epidemic control. In a game-theoretic model, individuals are assumed to be rational and rational individuals may themselves take mitigating measures. In such a set-up, increased social distancing reduces likelihood of infection but also comes with a cost in the form of reduced freedom, convenience, earning potential, and so on. Reluga (2010) considers a differential game capturing precisely these trade-offs. Valdez et al. (2012) allow for the possibility that individuals can identify infected neighbours and temporarily interrupt contact with them.

**Incorporating social distancing policies into an economic model.** Lastly, while we have focused on the benefits to public health of social distancing, this is far from the full story. Any amount of social distancing brings with it economic cost and, as evidenced by recent economic data, the policy responses to the COVID-19 outbreak have had an enormous effect on the global economy.^19^ Given that, as of time of writing, the mass roll-out of a successful vaccine to COVID-19 is potentially far on the horizon, some form of relaxed social distancing measures need to be considered.^20^ A richer model would also include the effect that social distancing has on economic output, and subsequently incorporate the interdependent relationship between output and public health (Bloom et al. 2004; Weil 2007). Such a model would allow policy makers to consider the full trade-off between absolute lockdown (everyone safe from exposure, economic activity greatly reduced) versus no lockdown of any kind (nobody safe from exposure, economic activity is maximal).

Lastly, we wish to reiterate that the framework in this paper considers the benefits to social distancing when it is the only policy available tool available. Clearly, this is not realistic. In real-world epidemics, policy makers have an array of tools available. While understanding how one policy works in isolation is a useful theoretical exercise, it is important to understand how the full set of policy tools complement each other in order to successfully operationalise a pandemic response plan.

Gregory Gutin has nothing to disclose. Sung-Ha Hwang has nothing to disclose. Tomohiro Hirano has nothing to disclose. Philip R Neary has nothing to disclose. Alexis Akira Toda has nothing to disclose.

## Footnotes

1 The modelling epidemics on networks is well-established. See for example Pastor-Satorras and Vespignani (2001) and Moreno et al. (2002). Kiss et al. (2017) is a recent textbook treatment.

2 Note that by ‘social distancing’ we mean limiting the number of social interactions in which an individual partakes. We do not mean individuals maintaining a predetermined physical distance throughout a social interaction as is sometimes intended. Our measure is adapted from the game-theoretic model of Netflix Games (Gerke et al. 2019; Gutin et al. 2020). There, the purchaser of a product may only share with a limited number of friends. Here, the carrier of an infectious pathogen is limited in the number of friends that they may interact with.

3 All replication files are available at https://github.com/alexisakira/COVID19_network.

4 To ensure we are comparing like-for-like, we fix it such that all networks are connected and possess the same average degree. This means that it is the way in which the societies are organised, and not the overall level of interaction, that is being varied.

5 The mathematical machinery underpinning our model is adapted from the game-theoretic model of Netflix Games introduced in (Gerke et al. 2019; Gutin et al. 2020). In a Netflix Game, each individual can be a Driver, *D*, who purchases Netflix, or a Passenger, *P*, who free-rides on the purchase of neighbours, and the capacity function determines how many neighbours a Driver may share with. The Nash equilibria of a Netflix Game are characterised by a novel kind of spanning subgraph referred to as *DP*-*Nash subgraph*. In the current environment, the capacity function dictates how many neighbours each individual may visit on a daily basis when a social distancing measures is in place.

6 A few things to note. First, technically we should write that vertex *i* selects $\min \left\{ \kappa (i), d(i)\right\}$ neighbours since a vertex cannot select more neighbours than it has. Second, note that the model reduces to the standard networked SIR model when $\kappa (i) = d(i)$ for all $i \in V$. Third, when $0\le \kappa (i) < d(i)$ for some $i \in V$, there are $$\left( {\begin{array}{c}d(i)\\ \kappa (i)\end{array}}\right) $$ ways for *i* to select a subset of neighbours of size $\kappa (i)$. We assume a uniform distribution over the likelihood of choosing each subset of neighbours which implies no individual has a ‘favoured neighbours’. This can of course be relaxed.

7 There are continuous-time versions of the SIR model. In such environments, the parameters $\beta$ and $\gamma$ are interpreted as rates or flows and are not probabilities constrained to lie in (0, 1). Of course, when running simulations, one must discretise these continuous-time models.

8 The majority of theoretical papers on disease spread on networks focus on scenarios in which the disease will become endemic with probability 1 or will die out with probability 1. Lančić et al. (2011) categorise where in $(\beta , \gamma )$ space both these events occur with positive probability.

9 Here, we have assumed that the policy is constant over the window $\left\{ 1, \ldots , T\right\}$. If the policy is allowed to vary with time, as in Sect. 5.3, we simply get a sequence of policies indexed by time, $\left\{ \kappa _{1}, \kappa _{2}, \ldots , \kappa _{T}\right\}$.

10 Zhaoyang et al. (2018) find that the average number of daily interactions for adults is 12 although it varies with age. Our choice of $\bar{d} = 10$ is made so as to be in line with this.

11 These epidemic parameters may change with the accumulation of new scientific knowledge. Some of the parameters are updated at the Center of Disease Control and Prevention (CDC) website at https://www.cdc.gov/coronavirus/2019-ncov/hcp/planning-scenarios.html. As of September 2020, the best estimate of ${\mathcal {R}}_0$ is 2.5, with a possible range of 2.0–4.0.

12 We thank an anonymous referee for bringing this important point to our attention.

13 Many interesting policy experiments stipulate that different individuals face different social distancing constraints. We study one such scenario in Sect. 5.2 and propose others in Sect. 6.

14 Caveat: as per Footnote 6, when we say that ‘all vertices face constraint $N_{\max }$’, in actuality every vertex *i* is assigned capacity $\kappa (i) = \min \left\{ N_{\max }, d(i)\right\}$.

15 We consider peak infection rate as it captures the worst-case scenario. This has been cited as a particularly important measure in the COVID-19 epidemic where it is often the excess numbers of sick individuals that strain a healthcare system (limited numbers of intensive care beds, ventilators, and personal protective equipment for medical professionals) that can be as important an issue to public health as any other factor.

16 We assume that in both countries 1% of individuals are infected. Of course, it is possible to address a host of other related questions. An example would be, ‘Suppose *x*% of individuals in Country *A* are infected while nobody in Country *B* is infected. What then is the lowest value of *x* such that if *x*% of individuals in Country *A* are infected then the reach of the epidemic will be both Country *A* and Country *B*?’.

17 It is further possible that an individual’s likelihood of recovery may be decreasing in the number of those currently infected. The reason being that most countries have a fixed stock of medical resources, so additional hospitalisations reduce the per patient allocation of resources, which once a threshold is met will have ramifications for quality of treatment.

18 In the European Union, it is not uncommon to see the following occur: if the state of the pandemic in Country A is deemed sufficiently safe by Country B, then Country B will allow a ‘travel corridor’ from Country A. In practice, a travel corridor from A to B means that Country B will allow visitors who have been to country A in the last 14 days; in our framework, it would mean allowing (possibly directed) connections from A to B.

19 Baker et al. (2020) document that the USA’s week-ending jobless claims numbers from late-March 2020 have been an order of magnitude higher than any seen previously. Moreover, these numbers were as a result of the lockdown policy and not the virus itself.

20 Dingel and Neiman (2020) estimate that only one-third of jobs in the USA can be done from home.
